# Ultraviolet photodetection of flexible ZnO nanowire sheets in polydimethylsiloxane polymer

**DOI:** 10.3762/bjnano.3.41

**Published:** 2012-05-02

**Authors:** Jinzhang Liu, Nunzio Motta, Soonil Lee

**Affiliations:** 1 School of Chemistry, Physics & Mechanical Engineering, Queensland University of Technology, Brisbane, 4001, Australia; 2 Division of Energy Systems Research, Ajou University, Suwon, 443-749, Republic of Korea

**Keywords:** permeable polymer, photoresponse, polydimethylsiloxane, UV photodetection, ZnO nanowires

## Abstract

ZnO nanowires are normally exposed to an oxygen atmosphere to achieve high performance in UV photodetection. In this work we present results on a UV photodetector fabricated using a flexible ZnO nanowire sheet embedded in polydimethylsiloxane (PDMS), a gas-permeable polymer, showing reproducible UV photoresponse and enhanced photoconduction. PDMS coating results in a reduced response speed compared to that of a ZnO nanowire film in air. The rising speed is slightly reduced, while the decay time is prolonged by about a factor of four. We conclude that oxygen molecules diffusing in PDMS are responsible for the UV photoresponse.

## Introduction

ZnO is a direct wide band gap semiconductor with a 3.37 eV gap and a high exciton binding energy of 60 meV at room temperature, which is promising for optoelectronic applications, including light-emitting diodes, laser diodes, and photodetectors for the ultraviolet (UV) spectral range [1–2]. ZnO nanostructures are particularly interesting as they bear superior properties compared to the bulk crystal. UV-light detection is one of the major applications of ZnO, and various UV photodetectors based on ZnO films or nanocrystals have been reported. It has been demonstrated that ZnO nanowires have high internal photoconduction gain and much stronger responsivity under UV-light illumination compared to the bulk film [3]. The UV photoresponse mechanism of ZnO nanowires is dominated by the adsorption and desorption of oxygen molecules [4]. In vacuum, ZnO nanowires show a prolonged UV photoresponse time and lowered responsivity [5]. So far, many UV photosensors have been made from ZnO one-dimensional nanostructures with various configurations, for sensing elements, such as single-nanowire devices [6], nanowire bridges [7], nanobelt network films [8], vertical nanowire arrays [9], and flexible nanowire sheets [10]. These nanostructures are normally exposed to air for measurement, neglecting the possible surface contamination caused by absorption of other molecules from the ambient atmosphere, which can degrade the UV-sensing performance of ZnO. For example, it has been demonstrated that the UV photoconduction of ZnO nanowires is degraded by the adsorption of water molecules in humid air [5,11]. Water molecules together with CO_2_ in a moist atmosphere bring about a slow chemical reaction with ZnO to form amorphous Zn(CO)_3_, which roughens the surface of the nanowires [12]. Therefore, for practical application the device encapsulation is essential to ensure a clean oxygen atmosphere around the nanowires. In this work we investigate the performance of polymer-embedded ZnO nanowires for UV photodetection, to avoid the need to use a gas cavity to load the nanowires, simplifying the device fabrication.

Recent experiments have demonstrated that coating ZnO nanobelts with UV-sensitive polymers enhances the UV photoconduction [13–14]. A thin layer (<100 nm) of such a polymer over ZnO nanocrystals produces excited states upon UV illumination. The excited states of the polymer molecules act as transition states facilitating electron hopping from the valence band of ZnO into the conduction band. However, the coating of such a polymer over ZnO nanowires must not be too thick because it would block the UV light. Moreover, carbon-based polymers undergo oxidation with ageing, and the degradation could be expedited by the absorption of UV light.

In this paper, we report results of experiments on UV photodetectors based on a thin sheet of ZnO nanowires embedded in polydimethylsiloxane (PDMS), which is an optically clear, UV-transparent silicone polymer that has been used in the fabrication of contact lenses, microfluidic devices, and stretchable displays. Due to its excellent gas permeability this polymer has been applied as a membrane for gas separation [15]. In our work, we combine free-standing thin-sheets of ZnO nanowires with the elastic material PDMS, suitable for developing bendable devices, which are of interest in current research.

## Results and Discussion

**Figure 1:**
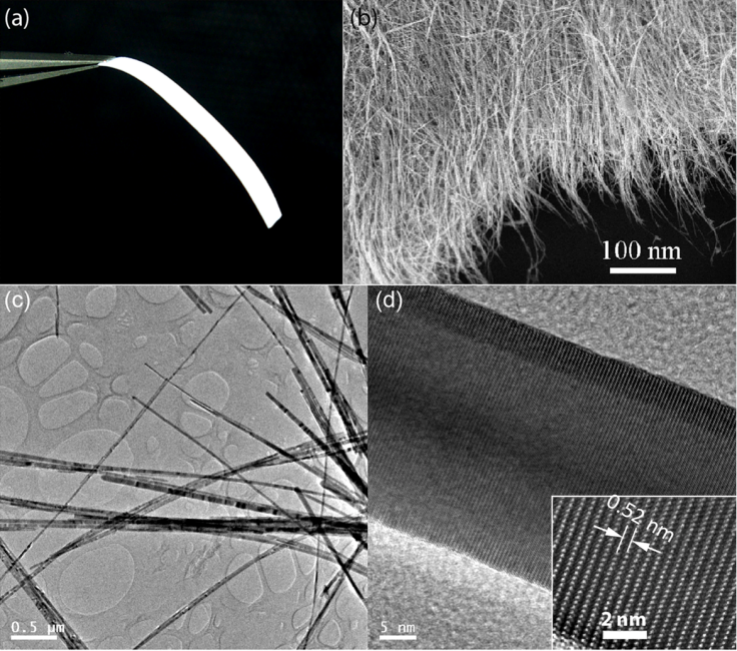
(a) An optical photograph of a strap-shaped ZnO nanowire film; (b) SEM image of the nanowire film; (c,d) TEM images of the nanowires.

ZnO nanowires were synthesized on a large scale by a modified carbothermal reduction method. Vaporized zinc and oxygen react at atmospheric pressure and result in a cotton-like product consisting of ZnO nanowires. The nanowires were processed into free-standing thin sheets, which can be cut by a blade into any shape [16]. The optical photograph in Figure 1a shows a strap-shaped nanowire sheet held by a tweezer. Scanning electron microscopy (SEM) observation reveals that these nanowires interdigitate to form a felt-like film (Figure 1b). Transmission electron microscopy (TEM) images of the nanowires are shown in Figure 1c and Figure 1d. The high-resolution TEM image in Figure 1d reveals that the nanowires grow along the [1] direction, as the distance between the (0001) planes is 0.52 nm. The nanowires are 20–60 nm in thickness and tens of microns in length. The high surface-to-volume ratio ensures a large surface adsorbance of gaseous molecules.

**Figure 2:**
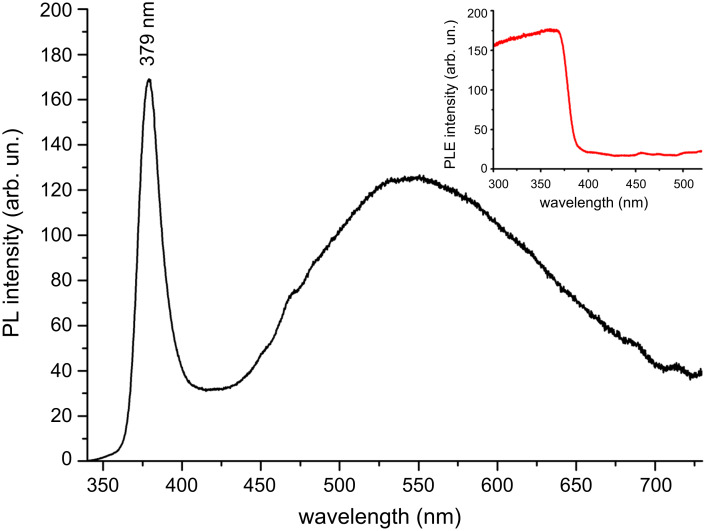
Room-temperature PL spectrum of the ZnO nanowire film. The insert is a PLE spectrum.

Figure 2 shows the room-temperature photoluminescence (PL) spectrum of the ZnO nanowires, at the excitation wavelength of 325 nm. The insert is a PL excitation (PLE) spectrum taken for the emission at 570 nm. The PLE spectrum indicates that these nanowires absorb UV light with a wavelength below 378 nm, whereas they are transparent to visible light. In the PL spectrum the sharp peak at 379 nm is ascribed to the near-band-edge emission of ZnO. The broad emission band in the visible range is related to some crystal defects as luminescent centres. In our method, the nanowires were grown quickly by vapour-phase reaction at low temperature, below 200 °C, and crystal defects are expected. Oxygen vacancies and antisite oxygen give contributions to the green PL emission around 520 nm; zinc vacancies and interstitial oxygen contribute to the blue PL emission (450–470 nm) [17].

**Figure 3:**
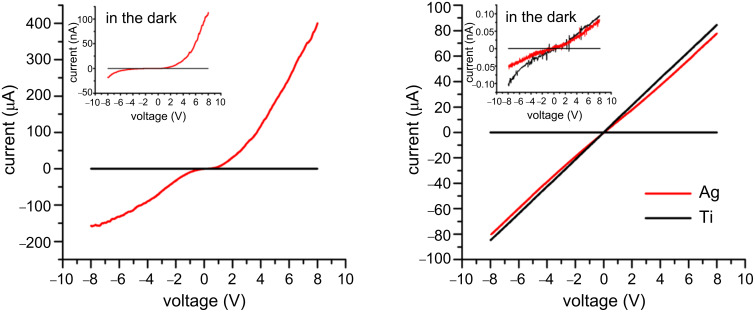
*I–V* curves of ZnO nanowire films under UV illumination and in the dark. (a) The device in PDMS with Ag–PDMS paste electrodes; (b) two devices in air, with vacuum-deposited metal electrodes (Ti or Ag).

We made devices based on the strap-shaped nanowire sheets (width 2 mm). To make electrode contacts, silver paste made by mixing Ag nanopowder and the viscous PDMS liquid was painted onto a nanowire film strap at two ends, leaving a 10 mm gap between the two electrodes. The whole device, with a Ag–ZnO–Ag structure, was embedded in PDMS for measurement. The PDMS liquid wets the ZnO nanowires well, such that after curing the interspaces between nanowires were filled with PDMS polymer, making the paper-like nanowire sheet more translucent. For comparison, we made two devices without embedding in PDMS. For these two devices we deposited metal electrodes (Ti or Ag) onto the strap-shaped ZnO nanowire films (2 mm width) by e-beam evaporation through a shadow mask, in order to rule out the influence of PDMS on the UV-detection performance. The gap between two opposite electrodes is 10 mm, which is the same as that of the device in PDMS. If Ag–PDMS paste were applied onto the ZnO nanowire film, the liquid PDMS would slowly spread out of the agglomeration of Ag nanoparticles and infiltrate the nanowire film due to capillarity. Figure 3a shows the current–voltage (*I–V*) curves of the device in PDMS, measured under UV-light illumination (312 nm, 30 mW·cm^−2^) and in the dark. The *I–V* curves of two other devices measured in air are plotted in Figure 3b. ZnO is naturally n-type with an electron affinity of 4.35 eV; the work functions of Ag and Ti are 4.26 eV and 4.33 eV, respectively. Under UV illumination Ti shows good ohmic contact on ZnO due to the extremely low barrier at the Ti/ZnO junction. The vacuum deposited Ag also shows nearly ohmic contact on ZnO (Figure 3b). However, for the contact of Ag paste on ZnO, the *I–V* curves measured under UV illumination and in the dark show rectifying features. Presumably, the Ag nanoparticles that we used to make the Ag–PDMS paste were spontaneously oxidized due to ageing. The surface Ag_2_O layer, a narrow bandgap semiconductor, added an extra barrier for electrons flowing between Ag nanoparticles and ZnO nanowires, resulting in the nonlinear *I–V* curves in Figure 3a. The *I–V* curve of the device in PDMS measured in the dark, plotted in the insert in Figure 3a, shows that at a forward bias of 8 V the current is only 0.11 µA, whereas under UV light illumination the current reaches 405 µA at 8 V, ~3650 times larger. However, for the two devices measured in air, the current values at 8 V are around 85 µA for the device with Ti electrodes, and 78 µA for the device with Ag electrodes. Previously, we studied the UV photoconduction of ZnO nanowire films with different PL properties, i.e., defect contents. The UV photocurrent of those ZnO nanowire films (2 mm wide and 10 mm long) with vacuum deposited Ag electrodes was in the range of 13–90 μA at 8 V when measured in air [10]. In this work, the UV photocurrent of the device with Ag paste electrodes, embedded in PDMS, exceeds 400 μA at 8 V. Even at a reverse bias of −8 V, the current is 160 μA, much higher than that of the device without PDMS. In the dark, the current values of the device in PDMS are 110 nA at 8 V and 18 nA at −8 V. However, the dark current of the ZnO nanowire films exposed to air is extremely low, about 0.1 nA at 8 V (see the insert in Figure 3b), indicating considerable adsorbance of oxygen on the nanowire surfaces. Hence, both the UV photoconduction and dark conduction of the nanowire film can be enhanced by PDMS coating. The responsivity of the device, defined as the photocurrent per unit of incident optical power, is determined by the UV photoconductivity of the ZnO nanowires. From the *I–V* curves we can deduce that the PDMS coating over ZnO nanowires results in an approximately fivefold enhancement in the responsivity of the device.

**Figure 4:**
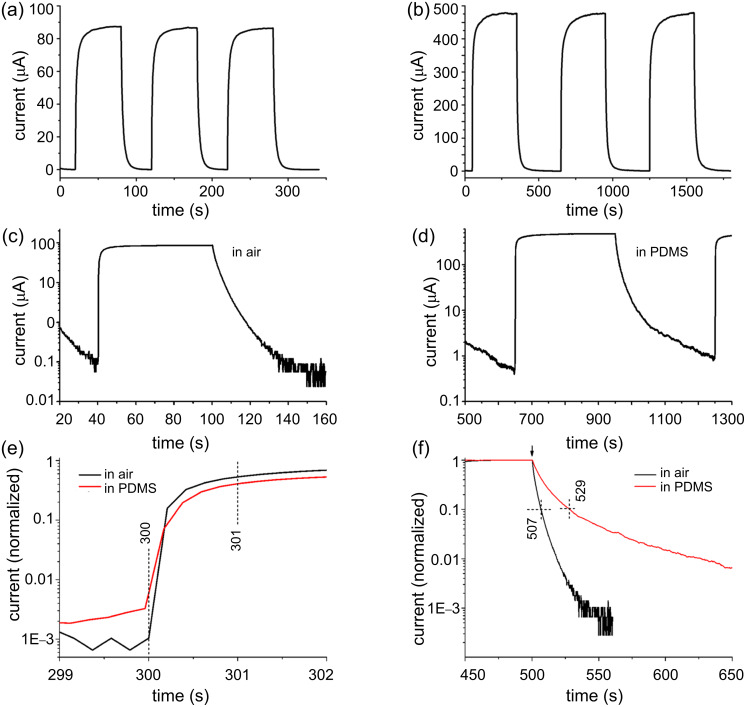
(a,b) UV photoresponse characteristics of ZnO nanowire sheets in air and in PDMS, respectively. The electrode of the device in air is vacuum-deposited Ti, and that of the device in PDMS is Ag–PDMS paste. The bias was set at 8 V for both devices; (c,d) UV photoresponse characteristics of the two devices with the current on a logarithic scale; (e and f) comparison of the UV-photocurrent rising and dark-current decay of the nanowire films in air and PDMS, respectively, with the current normalized by the maximum.

Figure 4 shows the UV-photoresponse characteristics of two devices based on ZnO-nanowire sheets with the same dimensions. The device with vacuum-deposited Ti electrodes was exposed to air, and the other one with Ag paste electrodes was embedded in PDMS. Both were measured at 8 V with the UV light switched on and off periodically. Figure 4a and Figure 4b show the UV photoresponse characteristics of the devices in air and in PDMS, respectively. The current–time curves depicting the UV photoresponse are further plotted in Figure 4c and Figure 4d on a logarithmic scale. The maximum photocurrent of the device in air is 86 μA, while that of the device in PDMS reaches 476 µA. The PDMS coating over ZnO nanowires leads to an enhancement of the UV photosensitivity and prolongs the decay time. The rise of the photocurrent and the dark current decay of the two devices are compared in Figure 4e and Figure 4f, respectively, with the current normalized to the maximum. Upon the switching on of the UV light, the current through the nanowire film in PDMS rises fast with a speed equivalent to that in air. Within one second the current reaches half of the maximum seen upon UV illumination. We define the dark current decay time as the time taken for the current to decay to 10% of the peak value. For the nanowire film in air the decay time is about 7 s, as shown in Figure 4f, whereas for that in PDMS the decay time is 29 s, i.e., about four times longer. Previous reports on ZnO nanowires that had gone through a lithography process involving polymers for making the electrodes [18–22], demonstrate a slow UV photoresponse compared to those that had never been in contact with polymers [7,9–10]. Surface contaminations from photoresist or electron-beam-resist polymers are probably responsible for the reduced response speed. The photoresponse speed of our ZnO nanowire film in PDMS is equivalent or even faster than for those contaminated by polymers from lithography processes, suggesting that ZnO nanowires in PDMS may be adequate for applications that do not require particularly high speeds.

The photoresponse of ZnO in air is known to be governed by the adsorption (in the dark) and desorption (under UV illumination) of oxygen molecules. In the dark, oxygen molecules adsorbed on the surface of ZnO nanowires capture free electrons of the n-type semiconductor: O_2_(g) + *e*^−^ → O_2_^–^(ad). This produces a depletion layer near the nanowire surface, resulting in an upward band bending near the surface. Due to the large surface-to-volume ratio, the adsorption of O_2_ significantly reduces the conductivity of the nanowires. UV-light illumination with a photon energy higher than *E*_g_ generates electron–hole pairs in the ZnO. Holes migrate to the surface along the potential slope created by the band bending and recombine with O_2_-trapped electrons, thus releasing oxygen from the surface: O_2_^–^(ad) + *h*^+^ → O_2_(g). The heavily populated electrons in the conduction band enhance the conductivity of the ZnO nanowires.

**Figure 5:**
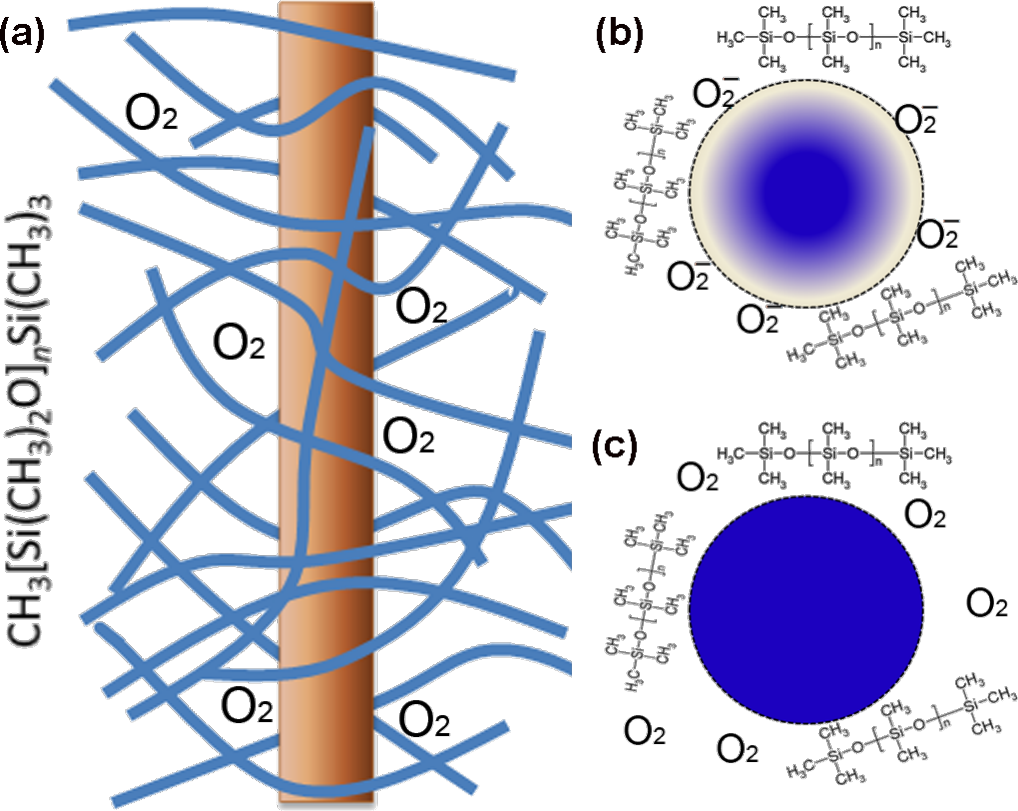
(a) Illustration of a ZnO nanowire in cross-linked PDMS. The blue lines represent PDMS molecule chains; (b) Illustration of a nanowire in PDMS without UV illumination, with oxygen adsorbed on parts of the surface. The colour gradient across the cross-section indicates a depletion layer near the surface; (c) Under UV illumination the oxygen molecules desorb from ZnO and diffuse into the molecule network.

The UV photoresponse of ZnO nanowires in PDMS can be explained with the help of Figure 5. After polymerization, the PDMS molecule chains of CH_3_[Si(CH_3_)_2_O]*_n_*Si(CH_3_)_3_, where *n* is the number of repeating monomer [SiO(CH_3_)_3_] units, form a network polymer structure that is highly permeable. PDMS has a high oxygen permeability due to the large free volume from the flexibility of the siloxane (–SiO–) linkages; the oxygen concentration in PDMS has been reported as 2 mM [23]. The diffusion coefficient of oxygen in PDMS is reported as 3.55 × 10^−5^ cm^2^·s^−1^ [24]. Therefore, entangled PDMS molecule chains and dissociated oxygen molecules surround the ZnO nanowires, as illustrated in Figure 5a. The interaction between ZnO and the cross-linked PDMS is believed to be of the van der Waals type. Charge interaction between the two materials is unlikely to occur, based on the fact that the PL properties of ZnO nanowires in air and embedded in PDMS are very similar. In the dark the current of the nanowire film in PDMS reached 110 nA at 8 V as shown in Figure 3a, whereas the dark current of such a nanowire film exposed to air was only 0.1 nA at 8 V, as reported in our previous work [10]. This means that the nanowires in PDMS have a smaller surface available for the absorbtion of oxygen molecules. In fact, when a ZnO nanowire is embedded in cross-linked PDMS, it is conceivable that only the nanowire surface areas that are not in contact with PDMS adsorb oxygen molecules diffusing through the rubbery polymer (Figure 5b). Upon UV illumination the holes in the valence band combine with O_2_-trapped electrons, releasing the surface O_2_ and enhancing the photoconduction (Figure 5c). The desorbed O_2_ diffuse into PDMS molecule networks, and are slowly re-adsorbed onto the ZnO nanowires after the UV light is switched off. PDMS molecule chains hamper the migration of oxygen molecules towards the ZnO nanowire surface, leading to the slow decay of the dark current. It is worth mentioning that the UV photoresponse speed of ZnO nanowires in an oxygen atmosphere is also dependent on the gas pressure, i.e., oxygen concentration: The lower the pressure the slower the UV photoresponse speed [4]. A number of silicone and polysulphone polymers have been studied for gas separation membranes [25–26]. Hence, we speculate that ZnO nanowires embedded in a gas-permeable polymer with higher oxygen permeability would have a faster UV photoresponse. The UV penetration depth in ZnO is less than 100 nm, whereas the thickness of the nanowire film is several micrometers, indicating that the contribution to the photoconduction of a nanowire sheet comes mostly from the top layers. With PDMS (refractive index ≈ 1.5) filling the interspaces between nanowires, the paper-like ZnO nanowire film becomes more translucent to the naked eye, facilitating UV-light scattering to a deeper level in the nanowire sheet. Therefore more nanowires will receive UV photons, leading to the enhanced responsivity of the nanowire sheet in PDMS.

## Conclusion

In summary, we demonstrated that ZnO nanowires embedded in PDMS polymer show great promise for UV detection. Our device based on a free-standing nanowire sheet in PDMS is bendable and facile to fabricate. The rubbery PDMS, which is gas permeable, not only provides protection to ZnO nanowires but also enables them to adsorb oxygen molecules diffusing through the polymer. Hence the UV-photoresponse mechanism of the nanowires in PDMS is analogous to that of nanowires exposed to an oxygen atmosphere. The PDMS coating over ZnO nanowires leads to an enhancement of responsivity, but at the expense of speed compared to the same uncoated nanowires. However, the response speed of the ZnO nanowire film in PDMS is still acceptable and in the range previously reported for ZnO nanowire films exposed to air. Our study opens a way for making robust UV photosensors incorporating ZnO nanowires embedded in gas-permeable polymers.

## Experimental

The ZnO nanowires were synthesized within a horizontal tube furnace (inner diameter 5 cm) at atmospheric pressure without the use of any catalyst. Mixtures of ZnO and graphite powders (2–3 g), at a weight ratio of 1:1, were heated to 1100–1200 °C, and the vaporized growth species were transported by a gas flow that consisted of 1000 sccm N_2_ and 30 sccm O_2_. Cotton-like white products were deposited in the low-temperature (between ca. 200 °C and room temperature) region. We placed a curved aluminium foil against the inner wall of the quartz tube for collection of the nanowire product [17]. Thin sheets of ZnO nanowires were fabricated by a simple filtration method. First, a ZnO-nanowire suspension, with a concentration of 1 mg·mL^−1^, was prepared by ultrasonic dispersion of the nanowires in isopropanol. Subsequently, the ZnO-nanowire suspension was vacuum filtered through a porous anode aluminum oxide (AAO) membrane, diameter of 4.3 cm and pore size of 200 nm, purchased from Whatman Co. Then the network film of ZnO nanowires on an AAO membrane was dried in air at 100 °C for 1 h. Finally, the thin sheet of ZnO nanowires was detached from the membrane filter.

**Figure 6:**
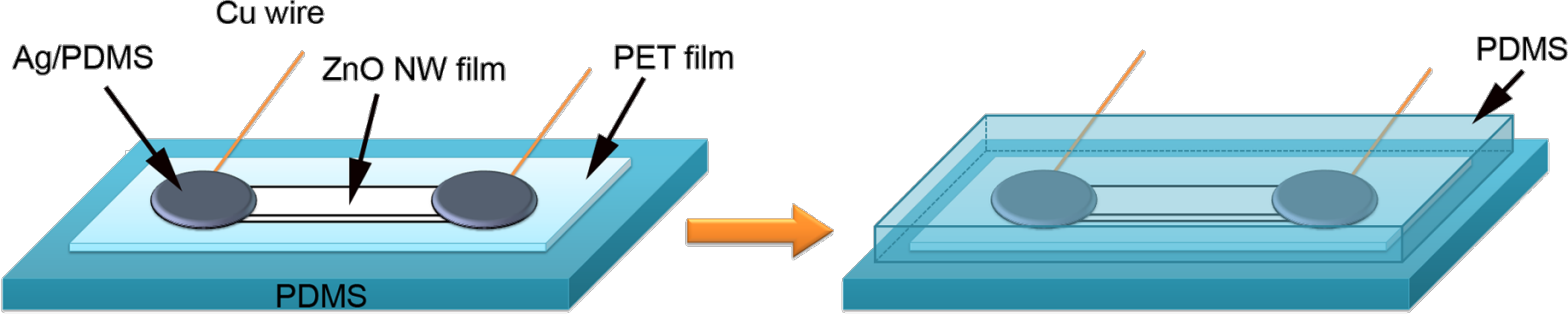
Illustration for the device fabrication process.

PDMS polymer was prepared by mixing the viscous PDMS liquid (Sylgard 184) and the cross-linking agent at a mass ratio of 10:1. The mixed PDMS was poured into a polystyrene Petri dish and dried in vacuum for 5 h to form a rubbery PDMS film. The PDMS liquid was also used for making the silver paste by blending with Ag nanopowder (diameter ca. 100 nm). The device fabrication process is illustrated in Figure 6. First, a poly(ethylene terephthalate) (PET) film is placed onto a PDMS layer. Onto the PET film a strap-shaped ZnO nanowire film is laid down and then silver paste is painted at the two ends. At each end a thin copper wire is in contact with the silver paste. We wait for 5 h until the PDMS blended with Ag nanoparticles is cured. Afterwards, we put the device into a plastic Petri dish and pour PDMS liquid onto the nanowire film to thoroughly seal the device, followed by a vacuum drying process for cross-linking of the polymer. The thickness of the PDMS layer above the ZnO nanowire film can be controlled by adjusting the volume of PDMS liquid in the Petri dish. In this work the thickness of the PDMS layer above the ZnO nanowire film was about 2 mm.

For the devices to be measured in air, we deposited 100 nm thick metal electrodes (Ti or Ag) onto the strap-shaped ZnO nanowire sheets (width 2 mm) by e-beam evaporation through a shadow mask. The electrode area was 3 mm long and 2 mm wide, as defined by the hollow pattern in the shadow mask. The gap between two opposite electrodes was 10 mm.
